# Latest Advances in Intersphincteric Resection for Low Rectal Cancer

**DOI:** 10.1155/2020/8928109

**Published:** 2020-07-20

**Authors:** Yifan Xv, Jiajun Fan, Yuan Ding, Yang Hu, Yingjie Hu, Zhengjie Jiang, Qingsong Tao

**Affiliations:** Department of General Surgery, Zhongda Hospital, School of Medicine, Southeast University, 87 Dingjiaqiao Road, Nanjing 210009, China

## Abstract

**Background:**

Intersphincteric resection (ISR) has been a preferable alternative to abdominoperineal resection (APR) for anal preservation in patients with low rectal cancer. Laparoscopic ISR and robotic ISR have been widely used with the proposal of 2 cm or even 1 cm rule of distal free margin and the development of minimally invasive technology. The aim of this review was to describe the newest advancements of ISR.

**Methods:**

A comprehensive literature review was performed to identify studies on ISR techniques, preoperative chemoradiotherapy (PCRT), complications, oncological outcomes, and functional outcomes and thereby to summarize relevant information and controversies involved in ISR.

**Results:**

Although PCRT is employed to avoid positive circumferential resection margin (CRM) and decrease local recurrence, it tends to engender damage of anorectal function and patients' quality of life (QoL). Common complications after ISR include anastomotic leakage (AL), anastomotic stricture (AS), urinary retention, fistula, pelvic sepsis, and prolapse. CRM involvement is the most important predictor for local recurrence. Preoperative assessment and particularly rectal endosonography are essential for selecting suitable patients. Anal dysfunction is associated with age, PCRT, location and growth of anastomotic stoma, tumour stage, and resection of internal sphincter.

**Conclusions:**

The ISR technique seems feasible for selected patients with low rectal cancer. However, the postoperative QoL as a result of functional disorder should be fully discussed with patients before surgery.

## 1. Introduction

Colorectal cancer is the third largest cancer in the world. The low rectum is usually defined as the lower third of the rectum within 5–6 cm from the anal verge [1] or 2 cm above the dentate line. Low rectal cancer refers to the cancer located at the lower edge of the cancer less than 5 cm from the anal edge. Standard surgical treatment for massively invasive rectal carcinoma located within 5 cm from the anal verge is abdominoperineal resection (APR) conventionally. However, the permanent colostomy often results in poor quality of life (QoL) and severe psychological trauma. The intersphincteric resection (ISR), which was first proposed by Schiessel in 1994 for more distal location, combines rectal removal with part or the whole of internal anal sphincter excision and restores by hand sewn coloanal anastomosis [2]. ISR has nowadays been increasingly recognized to achieve a safe distal resection margin (DRM), which can be as small as 1–2 cm [3, 4].

## 2. Intersphincteric Resection

**Figure 1:**
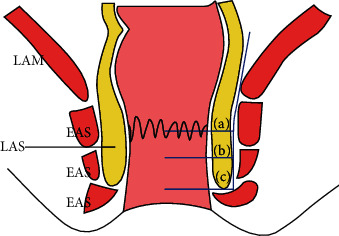
Type of ISR according to amount of excision of the internal anal sphincter. (a) Partial ISR. (b) Subtotal ISR. (c) Total ISR. EAS: external anal sphincter; IAS: internal anal sphincter; LAM: levator ani muscle.

**Figure 2:**
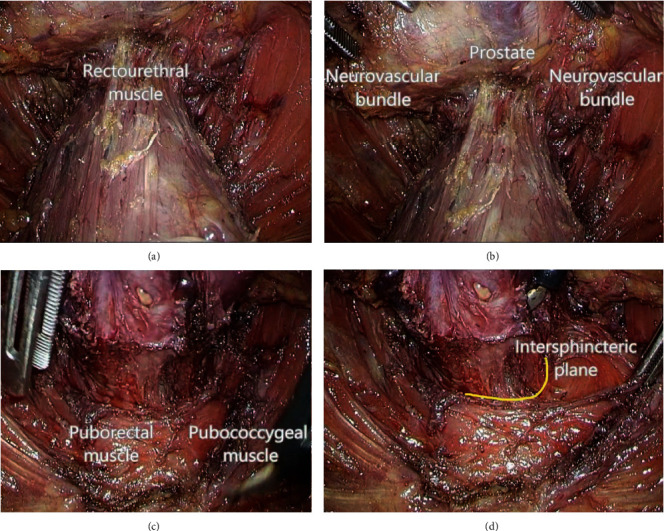
Laparoscopic procedure. (a) Dissection on the anterior side of the rectum. (b) Protection of prostate and neurovascular bundle. (c) Separation of puborectal muscle and the rectum. (d) Dissection of the intersphincteric plane.

The intersphincteric resection (ISR) is performed by ligation and dissection at the root of inferior mesenteric artery and vein, with total mesorectal excision (TME) and lateral lymph node dissection. Beyond the inferior margin of the mesorectal envelope, the puborectal muscle is separated from the rectal tube and the intersphincteric groove is entered wherever possible from above. Incision of the intersphincteric space between the internal and external anal sphincter is performed from the posterior side of the rectum by transecting the hiatal (anococcygeal) ligament [5]. To incise the internal sphincter deeply, a circumferential dissection is constructed on the anal mucosa at the level of dentate line in partial-ISR, between the dentate line and intersphincteric groove in subtotal-ISR, and at the intersphincteric groove in total-ISR [5] (see Figure 1), which depends on the lower border of the tumour so as to give a 1 cm clearance distally. The intersphincteric plane is developed circumferentially towards the cranial end to meet the plane from the abdominal dissection [6] (see Figure 2). The autonomic pelvic nerve is reserved, and a colonic J-pouch is used as a reservoir to manufacture a 5 cm short pouch for anastomosis in ISR [7]. In contrast, a coloplasty pouch is designed to interrupt antegrade colonic peristalsis and as an option when the pelvis is too narrow to permit a bulky colonic J-pouch anal anastomosis and the descending colon is too short to reach the anus [8]. Comparison of the stool frequency, the use of antidiarrheal medication, and continence have confirmed no significant differences between treatment with coloplasty pouch and colonic J-pouch. Patients' perceptions measured by the Fecal Incontinence Quality of Life Scale and WIS Score are also equivalent between the two techniques [9]. A crucial prerequisite is a meticulous preoperative evaluation of local tumour spread with rectal magnetic resonance imaging excluding infiltration of the external sphincter [10]. Although ISR was initially applied to treat inflammatory bowel disease, its performance in anal preservation for low rectal cancer has been supported by many studies [11–13]. Molnar et al. [12] followed up 20 patients with low rectal cancer for one year after ISR, discovering that the short-term survival rate was 100% and the median Wexner score of all patients was less than 10. Moreover, signs of local recurrence were absent, with antigen levels remaining within the reference ranges, and the incidence of complications like wound infection and postoperative pain within one year after ISR was lower than that of APR. Comparing 72 rectal cancer patients with ISR or APR for 5 years of follow-up, Molnar et al. [13] demonstrated that the 5-year overall survival rate was not influenced by surgical techniques while patients with ISR had better quality of life (QoL) postoperatively. ISR has been a feasible option to low rectal cancer patients who have a strong desire for anal preservation.

Laparoscopic intersphincteric resection (LISR) and robotic ISR (RISR) have been widely used with the proposal of 2 cm or even 1 cm rule of distal free margin and the development of minimally invasive technology in recent years [14]. Compared with the open intersphincteric resection (OISR), LISR and RISR reveal remarkable advantages such as less operation time, less blood loss, less postoperative complications, better pathological outcomes, and better survival results.

The gold standard of rectal cancer surgery is total mesorectal excision (TME) with improved survival and reduced local recurrence. TME involves precise excision of the entire rectum and pararectal lymph nodes en bloc, within an oncologic package termed the “mesorectal envelope” [15]. For patients with rectal cancer, the adequacy of the distal margin is dependent on both the risk for intramural tumour spread and the distal mesorectal lymphatic spread. Tumour cell deposits within mesorectal lymph nodes have been identified up to 5 cm distal to the inferior aspect of the tumour, underlining the need to adhere to the principles of TME and engendering the concept of tumour-specific mesorectal excision (mesorectal transection 5 cm distal to the inferior border of the tumour) for more proximal rectal cancers. Through TME, ISR acquires sufficient tumour excision, preservation of the sphincter function, and pathologically negative margin as well. Compared with APR, ISR possesses adequate distal resection margin (DRM), sufficient circumferential resection margin (CRM), and better anal function without permanent colostomy [16, 17]. A laparoscopically accomplished coloplasty pouch is easier to anastomose to the anus without tension, and complete laparoscopic TME with ISR is considered to decrease the surgical invasiveness without any additional abdominal incisions except those created for the laparoscopic port sites.

## 3. Preoperative Chemoradiotherapy

Preoperative chemoradiotherapy (PCRT) can reduce tumour volume and ensure negative CRM, which is often perceived as the standard preoperative treatment strategy for low rectal cancer patients with ISR. PCRT enables the DRM to be decreased to 5–10 mm [18], and a DRM of 10 mm is deemed to be safe and reasonable for anal preservation [17, 19]. Moreover, PCRT also makes a contribution to the decrease of local recurrence.

ISR following PCRT has been proved to be a valid alternative for ultralow rectal cancer patients who are reluctant to receive APR [20, 21]. Kawai et al. [22] reported a 67-year-old man with locally advanced rectal cancer accompanied by right lateral lymph node metastasis. After 6 courses of neoadjuvant chemotherapy with capecitabine, oxaliplatin, and irinotecan, remarkable tumour shrinkage was observed and the patient underwent safety ISR along with lateral lymph node dissection eventually. Okada et al. [23] also reported a 62-year-old man with a 5 cm locally advanced rectal cancer in the lower rectum close to the anal with mesorectal lymph node metastases. ISR with diverting loop ileostomy was performed as an anal-preserving surgical procedure after 12 courses of capecitabine plus oxaliplatin and bevacizumab, and no remnant tumour in the rectum or lymph node metastasis was found upon the pathological examination of resected specimens. No signs of recurrence were found at 12 months post operatively. Moreover, LISR with intraoperative radiotherapy using low-energy X-rays provides an opportunity of anal reservation for patients with late-stage ultralow rectal cancer. Wang et al. [24] reported a 53-year-old female patient with rectal adenocarcinoma which was 4 cm × 3 cm, located 2 cm away from the anus and invaded the levator ani muscles. Low-energy X-ray radiotherapy was applied during LISR, and no complications, anal dysfunction, and local recurrence occurred during follow-up.

Although many studies have proposed that PCRT makes anal preservation possible and improves prognosis, PCRT is inclined to spawn harm to anorectal function and patients' QoL, especially for patients with deep tumours. The main pathogenesis of anorectal function lesion induced by pelvic radiotherapy is anorectal irradiation. Radiation may injure the myenteric plexus and inhibit impulse conduction which has a bearing on anal sphincter lesion and fibrosis [25, 26]. Furthermore, PCRT has been demonstrated as a critical risk factor for postoperative anal dysfunction [27], which is probably connected with pelvic ischemic changes and fibrosis [28]. Significant reductions in anorectal maximum resting pressure and maximum crush pressure, deteriorations in the Wexner score, and disappearances of rectoanal inhibitory reflex are frequently observed in patients undergoing PCRT. Similarly, Gervaz et al. [29] discovered that PCRT prior to ISR was associated with lower urinary scores (gas, liquid, and solid incontinence) and more urinary dysfunction (aggregation and incomplete urination) than simple surgery. Nevertheless, Beraldo et al. [30] contradictorily raised that PCRT was not responsible for the deterioration of urinary symptoms. Furthermore, the implementation of ISR following PCRT is bound up with a high perioperative risk, especially for male patients with radiation colitis. Postoperative anastomotic leakage (AL) connected with chronic anastomotic stenosis [31] occurs more frequently and is more inclined to delay.

## 4. Complications

Common complications after ISR include anastomotic leakage (AL), anastomotic stricture (AS), urinary retention, fistula, pelvic sepsis, and prolapse. AL often brings about postoperative AS [32], poor anorectal function, and severe sepsis followed by death. It usually occurs 3-30 days after operation, with an incidence of 3%-20% [33, 34]. Delayed AL is reported to occur later than 30 days, and its incidence is 0.3-4.3%, accounting for about one third of all AL [35–38]. In addition to direct clinical consequences such as intra-abdominal or pelvic abscess, peritonitis, septicemia, prolonged hospitalization, and increased mortality, AL also induces pelvic organ dysfunction, anal dysfunction [39], local cancer recurrence, and increased cancer specific mortality [40].

Aggressive stage tumour, lymph node involvement, PCRT, and postoperative anemia are risk factors related to AL [41]. Besides the technical difficulties of anastomosis during operation, a predominant reason is the lack of blood supply after TME. The lower incidence of AL after colonic J-pouch compared to coloplasty pouch is conceived to stem from better proximal anastomotic blood supply. Another crucial factor for AL is poor pelvic drainage. Hematoma and seroma tend to gather and form in front of the sacrum after TME, consequently, infection and leakage into the anastomotic opening may promote rupture [42]. Kim et al. [43] discovered that the use of staplers increased the risk of AL, which was possibly owing to the fact that the larger diameter circular stapler caused the expansion of distal residual rectum and the attenuation of rectal wall, resulting in insufficient blood supply for the anastomosis. The colonic J-pouch with better vascularized end-to-side anastomosis shows functional superiority in reducing anastomotic dehiscence compared to the straight colonic-anal anastomosis [44]. It should be noted that PCRT is also considered to be an incentive for AL. Additionally, the incidence of recurrent AL is 25% [45] and it is often linked to hand-sewn anastomosis, ischaemia at the anastomotic site and a shorter interval between confirmation of healing and stoma closure.

Qu et al. [33] discovered that the incidence of AL after LISR (6.3%) was lower than that after APR (10%) in a meta-analysis owing to less bleeding and less lesion. The inflammatory reaction in LISR was also alleviated, which was conducive to the growth and healing of the anastomosis [46, 47]. It remains controversial whether protective stoma can reduce the incidence of AL. A multicenter randomized controlled study [48] by Matthiessen et al. has pointed out that protective stoma contributed to the decrease of AL while other studies [49, 50] have demonstrated that it bore no relation to the prevention of AL.

Anastomotic stricture (AS), a trigger for impaired function such as fecal urgency, incontinence, and bowel obstruction, is not a negligible complication [51], and its incidence ranges from 2.5% to 19.5% [52]. ISR with hand-sewn anastomosis, postoperative radiotherapy, male, and AL [53] are independent risk factors of AS. Stapled anastomosis is associated with a low incidence for including a full layer of anal stump and excessive dilatation of the anal canal during anastomosis. Radiation injury to the anorectum is inevitable, especially when radiotherapy is administered postoperatively. Radiation not only affects the anorectum and anastomosis but also induces proctitis, subsequent anastomotic fibrosis, and AS frequently [54]. Radiotherapy constantly brings about histologic alterations such as obliterative endarteritis, tissue ischemia, necrosis, and submucosal collagen deposition, consequently resulting in transmural fibrosis and formation of strictures [55, 56].

The incidence of urinary retention is reported to be 4.5%-41.0% [57]. Urinary retention not only induces pain and urinary tract infection but also promotes complications like venous thrombosis and hospital acquired pneumonia in elderly patients. It is associated with elderly patients, low tumour location, long operation time, excessive intraoperative transfusion volume, and early postoperative removal of catheter.

## 5. Oncologic Outcomes

Local control is one of the most important oncological objectives in surgery on patients with low rectal cancer. The local recurrence rate following sphincter-saving resections for low rectal cancer has been reported to range from 4% to 13% [58], and the 5-year local recurrence rate is not significantly different from that of low anterior resection (LAR) and ARP [7]. Local recurrence is related to pathological stage, local dedifferentiation, incision margin under microscope, postoperative anemia [41], and serum CA 19-9 level before operation [59]. Among them, CRM involvement is the most critical predictor. It must be emphasized that preoperative assessment and particularly rectal endosonography are important to select suitable patients with tumours confined to the rectal wall at least in its distal part and close to the sphincter [60].

For T1-T2 tumours, careful dissection and irrigation after closure of distal stump can be performed without PCRT. However, PCRT should be considered when resection margins are estimated to be insufficient for T3 tumours [61]. For T3 and/or lymph node-positive rectal cancers in general, PCRT has a tendency to reduce local recurrence, prolong overall survival (OS), and disease-free survival (DFS) better when compared with surgery alone [62]. Moreover, it is more effective for local control, less toxic, and sphincter preservation than postoperative therapy. In addition to the reduction of tumour volume and stage, it transforms tumour into ulcerative scar, thus refraining the spread of tumour during operation and subsequent local recurrence [58]. In the study of Hohenberger et al. [63], 35 of 53 patients (66%) received PCRT and 4 (11%) had local recurrence while local recurrence occurred in 7 (39%) of the 18 patients received blank control. However, it has been reported that the overall survival benefit of neoadjuvant chemotherapy has not increased, and the incidence of complications is even higher [60]. Akagi et al. [64] reported a low incidence of local recurrence (4.8%) and a five-year survival rate of 76-97% without neoadjuvant chemotherapy.

The 3-year overall survival (OS) and disease-free survival (DFS) in the study of Sakr et al. [65] were 91.4% and 79%, respectively. The OS in the ultralow anterior resection (ULAR) and ISR groups were 91.4% and 91.7%, respectively, while the DFS were 79% and 79.2%, without any significant difference. Park et al. [66] demonstrated that the two groups shared equivalent overall 3-year local recurrence rates when comparing RISR with LISR. The 3-year DFS rates were 89.6% in the robotic group and 90.5% in the laparoscopic group, respectively, without any significant difference. Unexpectedly, Portier et al. [67] investigated the oncologic outcomes of ultralow coloanal anastomosis with or without ISR for low rectal cancer, and no difference was noted in 5-year local recurrence rate and OS.

According to the meta-analysis of Zhang et al. [68], LISR had significant advantages over OISR like less operation time, less blood loss, less postoperative complications, better pathological outcomes, and superior survival results. The meta-analysis of Lee et al. [69] demonstrated that RISR shared comparable perioperative outcomes, prognosis, and survival results with LISR. Available data on comparison of perioperative (see Table 1) and oncologic (see Table 2) outcomes among OISR, LISR, and RISR are summarized from 10 articles [66, 70–78].

## 6. Functional Outcomes

One of the major issues for patients who undergo ISR for low rectal cancer is their QoL. Urgency, fecal incontinence (FI), frequent bowel movements, stool fragmentation, sense of incomplete evacuation, diarrhea and change in stool consistency have been highly noted. Anal dysfunction is considered to be closely related to age, PCRT, location of anastomotic stoma, growth of anastomotic stoma, tumour stage, and resection of internal anal sphincter, while the length of sphincteric resection (upper third or half) does not matter for the functional outcome [60, 79, 80]. PCRT is perceived to be linked to pelvic ischemic changes and fibrosis [28], and the main pathogenesis of anorectal dysfunction caused by pelvic radiotherapy is irradiation for myenteric plexus lesion and restrain of impulse conduction. It can also damage the anal sphincter and promote fibrosis [25, 26] directly. In addition, Shiokawa et al. [81] has confirmed the negative effect of extensive internal sphincter resection on anal function. Huang et al. [82] compared the anal function after LISR with OISR, and the result showed that the ratios of patients with good continence were 87.1% and 87.5%, respectively, during a mean follow-up of 52.0 months.

Clinical evaluation of anal function such as enhanced frequency of defecation, urgent defecation, difficulty in defecation, and FI has not been uniformed until now. The Jorge Incontinence Score, Kirwan Grade, and Wexner score are frequently used assessments. Anorectal manometry (ARM) is an objective evaluation and is widely employed for the differential diagnosis of resting and pressure reduction in patients with FI [83, 84]. High-resolution anorectal manometry (HR-ARM) is reliable for the evaluation of anal function after ISR objectively and accurately, and the high pressure zone and maximum resting pressure (MRP) may be useful preoperative predictors of severe FI after ISR. Kitaguchi et al. [85] found that maximum resting pressure and maximum squeeze pressure were significantly lower after ISR, and an elevated incidence of severe FI after ISR was independently related to a high pressure zone before ISR ≤ 3 cm and MRP before ISR > 60 mmHg. Lately, the fecoflowgrams calculated from defecography has also been a promising assessment of defecation after ISR [86]. Functional results after ISR reported by several studies [7, 11, 60, 80, 87–99] have been summarized in Tables 3 and 4.

Loss of the rectal inhibitory reflex, loss of the rectal storage function, the sphincter lesion caused by instrumental dilatation in stapling or hand-sewed anastomosis, and the disturbed function of the internal sphincter due to the autonomous nerve damage additionally contribute to the anal dysfunction. Regarding the bowel function, the fecal frequency after rectal surgery is dependent on the liquid stool present after the excretion of solid stool. Dysfunction of the anal sphincter allows the escape of liquid stool and the loss of the reservoir function cannot retain the liquid stool; therefore, the bowel function is presumably improved by solidifying the liquid stool of postoperative patients [100]. The nerve-sparing mesorectal excision helping to preserve the function of the internal sphincter has been recommended for better continence. Almost 50% of the patients suffer from an anterior resection syndrome after total or subtotal rectal resection with a straight colorectal or coloanal anastomosis, which describes the characteristic complaints of minor or major incontinence. The anastomosis with the colonic J-pouch has been proved to contribute to better continence in the short-term and long-term compared to the straight anastomosis [101]. Although ARM data show that colonic J-pouch shares identical volume with straight colostomy, the advantage of J-pouch lies in the alleviation of the intestinal motility and subsequent amelioration of the anal function [102]. The convenience of colonic J-pouch anastomosis in defecation frequency, urgency control, storage capacity, fecal incontinence and Wexner score has been reported to be optimized [103, 104].

Urinary dysfunction is another notable issue after rectal cancer surgery which may breed detrimental impacts on mental health and QoL of patients. Irritative nocturia and enhanced urinary frequency are the most common among urinary symptoms, 100% and 75%, respectively. Those symptoms are usually stemmed from mild pelvic neural injury compared to the obstructive symptoms [105, 106]. The sympathetic nerves from the superior hypogastric plexus and parasympathetic nerves from the pelvic splanchnic nerves control normal bladder and sexual function and are susceptible to injury during TME. Injury to sympathetic nerves may give rise to bladder instability and ejaculatory problems, while injury to the parasympathetic nerves may engender detrusor instability and erectile dysfunction [107]. Many studies have verified that LISR confer better genitourinary outcomes than open approach due to its amplified viewing angle for structure preservation and faster recovery [108, 109].

## 7. Conclusion

The complications, oncological outcomes, and functional outcomes appear to be acceptable after ISR. The ISR technique has been a feasible alternative to APR in selected patients with a low rectal cancer. However, it should be completely discussed with patients before surgery about the postoperative QoL as a result of functional disorder.

## Figures and Tables

**Table 1:** Comparison of perioperative outcomes among OISR, LISR, and RISR.

	OISR	LISR	RISR
*n*	264	586	226
Age (y)^∗^	58.7 (12.0)	60.7 (11.4)	58.6 (11.8)
Sex (male/female)	175/89	371/215	159/67
PCRT (%)	29.4	36.8	67.3
Distance from anal verge (cm)^∗^	4.3 (0.7)	3.7 (1.0)	3.3 (0.9)
Operation time (min)^∗^	296.6 (78.1)	288.1 (79.3)	308.1 (77.5)
Blood loss (ml)^∗^	210.7 (277.3)	99.4 (128.0)	128.3 (143.8)
Protective stoma (%)	30.0	44.1	53.3
Hospital stay (d)^∗^	16.0 (6.4)	11.4 (5.9)	10.9 (4.0)
Complication			
Wound infection	6	5	3
Anastomotic leakage	8	17	12
Anastomotic stricture	3	10	5
Bleeding	0	1	2
Ileus	7	15	12
Chylous ascites	1	2	0
Urinary retention	3	18	10
Pneumonia	8	6	0
Rectovaginal fistula	2	7	2
Intra-abdominal abscess	3	6	3

^∗^Values are mean (s.d.).

**Table 2:** Comparison of oncologic outcomes among OISR, LISR, and RISR.

	OISR	LISR	RISR
Tumour size (cm)^∗^	3.7 (1.6)	3.3 (1.7)	2.8 (1.8)
Lymph node retrieved^∗^	15.9 (9.8)	14.9 (9.1)	13.4 (7.7)
Proximal margin (cm)^∗^	15.6 (9.6)	18.7 (10.9)	20.7 (8.0)
Distal margin (cm)^∗^	1.7 (0.8)	1.7 (1.0)	1.4 (0.9)
Histopathological differentiation (%)			
Well	14.0	17.9	23.8
Moderate	76.6	74.7	66.7
Poor	9.4	6.0	6.0
Other type	0	1.4	3.5
Stage (%)			
pCR	0.5	2.0	6.0
I	35.4	36.0	39.3
II	26.4	27.6	23.8
III	34.9	30.6	23.8
IV	2.8	3.8	7.1
3-year LRR (%)	7.1	4.1	8.5
3-year DFS (%)	79.3	84.5	85.8
3-year OS (%)	84.5	89.3	94.2
5-year LRR (%)	2.0	6.6	8.7
5-year DFS (%)	71.0	76.3	80.6
5-year OS (%)	82.0	86.7	88.5

^∗^Values are mean (s.d.).

LRR: local recurrence rate; DFS: disease-free survival; OS: overall survival.

**Table 3:** Summary of functional results after intersphincteric resection.

	*n*	Wexner score^∗^		Kirwan grade				
		1 year	≥2 year	I	II	III	IV	V
Barisic 2011	45	3.6 (NR)	NR	NR	NR	NR	NR	NR
Butiurca 2019	60	8.6 (2.3)	7.3 (2.1)	NR	NR	NR	NR	NR
Chin 2006	10	NR	NR	3	2	3	2	0
Dumont 2013	14	11 (5.0)	NR	6	NR	NR	NR	8
Han 2009	35	NR	NR	15	10	9	1	0
Kawada 2018	28	10.4 (5.2)	7.9 (4.1)	NR	NR	NR	NR	NR
Kim 2016	62	7.9 (2.9)	5.6 (1.8)	44	11	4	3	0
Koyama 2014	37	NR	8.1 (4.8)	NR	NR	NR	NR	NR
Krand 2009	46	NR	NR	37	4	5	0	0
Kuo 2011	22	2.8 (NR)	NR	NR	NR	NR	NR	NR
Saito 2006	181	8.4 (4.5)	7.8 (4.2)	36	32	25	7	0
Vorobiev 2004	26	NR	NR	22	2	1	1	0
Yamada 2009	104	6.3 (5.1)	NR	44	31	26	3	0
Yamada 2019	990	7.2 (4.1)	NR	380	237	277	76	20
Zhang 2013	53	NR	NR	18	21	8	6	0

NR: not reported; NS: not sufficient data.

^∗^Values are mean (s.d.).

**Table 4:** Summary of functional results after intersphincteric resection.

	*n*	Mean stool frequency/24 h^∗^	Fecal urgency	Nocturnal defecation	Pad wearing	Intestinal transit regulators	Feces-flatus discrimination	Stool fragmentation	Antidiarrhea medication
Barisic 2011	45	1.8 (NR)	NR	NR	NR	NR	NR	NR	NR
Chamlou 2007	83	2.3 (1.3)	16	24	38	22	21	40	NR
Chin 2006	10	NS	5	NR	NR	NR	NR	NR	NR
Dumont 2013	14	NR	7	NR	NR	NR	NR	NR	5
Han 2009	35	2.7 (1.4)	11	NR	NR	NR	30	15	14
Kim 2016	62	4.0 (1.4)	NR	NR	NR	NR	NR	NR	NR
Koyama 2014	37	3.7 (2.2)	NS	NR	NR	NR	NR	NR	4
Krand 2009	46	NS	1	NR	NR	NR	2	7	0
Kuo 2011	22	4.7 (NR)	4	5	4	NR	NR	8	6
Vorobiev 2004	26	NS	1	NR	NR	NR	3	6	1
Yamada 2009	104	3.7 (1.6)	NR	NR	NR	NR	NR	NR	NR
Yamada 2019	990	5.0 (4.0)	NR	NR	NR	NR	NR	NR	NR
Yoo 2005	17	5.0 (2.0)	10	13	1	NR	NR	NR	NR
Zhang 2013	53	3.8 (1.3)	12	9	16	13	14	25	NR

NR: not reported; NS: not sufficient data.

^∗^Values are mean (s.d.).

## Data Availability

The data used to support the findings of this study are included within the article.
